# Evaluating Novel Agarose-Based Buccal Gels Scaffold: Mucoadhesive and Pharmacokinetic Profiling in Healthy Volunteers

**DOI:** 10.3390/pharmaceutics14081592

**Published:** 2022-07-30

**Authors:** Muhammad Ali Syed, Ghiyyas Aziz, Muhammad Bilal Jehangir, Tanveer A. Tabish, Ameer Fawad Zahoor, Syed Haroon Khalid, Ikram Ullah Khan, Khaled Mohamed Hosny, Waleed Yousof Rizg, Sana Hanif, Rabia Arshad, Muhammad Abdul Qayyum, Muhammad Irfan

**Affiliations:** 1 Department of Pharmaceutics, Faculty of Pharmaceutical Sciences, GC University Faisalabad, Faisalabad 38000, Pakistan; ma.pharmacist@hotmail.com (M.A.S.); syedharoonkhalid@gcuf.edu.pk (S.H.K.); ikramglt@gmail.com (I.U.K.); 2 Faculty of Pharmacy, The University of Lahore, Lahore 54590, Pakistan; pharmacist.sas22@gmail.com (S.H.); rabia.arshad@bs.qau.edu.pk (R.A.); 3 Department of Public Health, The University of Queensland, Brisbane 4076, Australia; ghiyyas.awan@yahoo.com; 4 Department of Medicine, Mohi-ud-Din Islamic Medical College, Mohi-ud-Din Islamic University, Nerian Sharif AJ&K 12080, Pakistan; mbj2094@gmail.com; 5 Division of Cardiovascular Medicine, Radcliffe Department of Medicine, University of Oxford, Headington, Oxford OX37BN, UK; tanveer.tabish@cardiov.ox.ac.uk; 6 Department of Chemistry, Government College University, Faisalabad 38000, Pakistan; fawad.zahoor@googlemail.com; 7 Department of Pharmaceutics, Faculty of Pharmacy, King Abdulaziz University, Jeddah 21589, Saudi Arabia; kmhomar@kau.edu.sa (K.M.H.); wrizq@kau.edu.sa (W.Y.R.); 8 Center of Excellence for Drug Research and Pharmaceutical Industries, King Abdulaziz University, Jeddah 21589, Saudi Arabia; 9 College of Pharmacy, University of Sargodha, Sargodha 40162, Pakistan; 10 Department of Chemistry, Division of Science & Technology, University of Education, Lahore 54770, Pakistan; hmaqayyum@ue.edu.pk

**Keywords:** industrial development, buccal mucoadhesive, agarose gels, local action, volunteer study, stability study

## Abstract

Agarose (AG) forms hydrocolloid in hot water and possesses a noteworthy gel strength. However, no reasonable scientific work on investigating the mucoadhesive character of AG has been reported. Therefore, the current study was designed to develop AG and carbopol (CP) based buccal gel scaffold for simultaneous release of benzocaine (BZN) and tibezonium iodide (TIB). Gels’ scaffold formulations (F1–F12) were prepared with varied concentrations (0.5–1.25% *w*/*v*) of AG and CP alone or their blends (AG-CP) using homogenization technique. The prepared formulations were characterized for solid-state, physicochemical, in vitro, ex vivo, and in vivo mucoadhesive studies in healthy volunteers. The results showed that mucoadhesive property of AG was concentration dependent but improved by incorporating CP in the scaffolds. The ex vivo mucoadhesive time reached >36 h when AG was used alone or blended with CP at 1% *w*/*v* concentration or above. The optimized formulation (F10) depicted >98% drugs release within 8 h and was also storage stable up to six months. The salivary concentration of BZN and TIB from formulation F10 yielded a C_max_ value of 9.97 and 8.69 µg/mL at 2 and 6 h (t_max_), respectively. In addition, the FTIR, PXRD, and DSC results confirmed the presence of no unwanted interaction among the ingredients. Importantly, the mucoadhesive study performed on healthy volunteers did not provoke any signs of inflammation, pain, or swelling. Clearly, it was found from the results that AG-CP scaffold provided better mucoadhesive properties in comparison to pure AG or CP. Conclusively, the developed AG based mucoadhesive drug delivery system could be considered a potential alternative for delivering drugs through the mucoadhesive buccal route.

## 1. Introduction

The oral drug delivery route is considered as the most preferable route owing to the maximum compliance and versatility of the dosage form accessibility in terms of localized and systemic mucosal/buccal delivery. However, owing to the involvement of numerous biochemical and physiological parameters in altering the pharmacokinetic aspects of various drugs, conventional drug delivery is still a big challenge [1]. Oral drug delivery is often associated with pre-systemic clearance issues which lead to poor absorption and bioavailability [2]. Therefore, challenges associated with oral as well as parenteral drug delivery prompted the need for exploring new mechanistic approaches for alternative routes [3]. Consequently, transmucosal routes of drug delivery offer discrete advantages over per-oral administration for systemic effect [4]. Amongst the several transmucosal routes, buccal mucosa has promising features in terms of controlled release, direct systemic circulation, and rapid uptake (as well as improved bioavailability) [5]. However, the main impediments faced by drugs through the buccal route are basically associated with the limited absorption area and mucoadhesion. Less mucoadhesion with respect to the buccal drug delivery is caused by continuous shedding of saliva followed by dilution of the drug [6]. We reported in our previous study that AG possessed poor mucoadhesive property when formulated in compressed mucoadhesive tablets as a monopolymer [7]. However, composite based tablets with CP revealed slightly improved mucoadhesion properties. Herein, this present research was focused on addressing the concerns by synthesizing smart composites mucoadhesive buccal gels through inclusion of biocompatible and mucoadhesive polymers [8,9]. Therefore, a novel strategy for evaluating the mucoadhesive potential of AG and CP based buccal gel scaffold for simultaneous release and synergistic uptake of BZN and TIB was developed and characterized. Gels scaffold formulations (F1–F12) were developed using different concentrations (0.5–1.25%, *w*/*v*) of AG and CP alone or their blends using homogenization technique. Furthermore, the optimized formulations were characterized based on physicochemical, in vitro, ex vivo, and in vivo experimentation. In addition, this study also aimed at improving the physiochemical properties within the polymeric matrix, thus providing better strength and swelling index. The impact of polymeric blend concentration was directly proportional to mucoadhesion strength and bioavailability [10]. Conclusively, the AG and CP polymeric blend based buccal gels possessed better mucoadhesive properties without inducing adverse clinical response on human volunteers [9,11]. Therefore, AG-CP gel composites could be justified as a prospective transporter in the case of the mucoadhesive buccal route.

## 2. Materials and Methods

### 2.1. Materials

Tibezonium iodide (Recordati^®^, Correggio, Italy) was received as a gift from Pacific Pharmaceuticals Ltd., Lahore, Pakistan. Benzocaine was generously donated by Remington Pharma, Pvt. Ltd., Lahore, Pakistan. Agarose of off white color (labelled gel strength >1200 g/cm^2^ and having a gelling temperature ranged between 35–37 °C) was sourced from bioWORLD^®^ (Visalia, CA, USA). Carbopol 934^®^, lactose, polyvinyl pyrrolidone, magnesium stearate and sucralose were kindly provided by Hoover Pharma Pvt. Ltd., Pakistan. Other chemicals such as sodium lauryl sulphate (SLS), sodium dihydrogen phosphate, acetonitrile, dimethyl sulfoxide (DMSO), triethanolamine (TEA) and o-phosphoric acid were used as received. Millipore^®^ (Burlington, MA, USA) filtered reverse osmosis (RO) water was used throughout this study unless otherwise mentioned.

### 2.2. Formulation Design

In the sustained release gel scaffold study, the polymeric design was similar to the smart mucoadhesive buccal gels composites study. However, the concentration of both polymers was exceeded the former. The concentrations of AG and CP for the fabrication of gels were studied in the range of 0.5–1.25% *w*/*v* in formulations F1–F8. However, the amounts were different as compared with contents when studied for compressed dosage form [7]. In contrast, the formulations F9–F12 contained the polymeric blend of AG-CP in equal concentrations ranging from 0.5–1.25% *w*/*v* (Table 1).

### 2.3. Formulation Technique

Briefly, the weighed mass of AG powder was dissolved in 100 mL solution of 0.05% *w*/*v* of propyl paraben in distilled water by heating at 95 °C which followed by cooling below 40 °C to form gels [12]. Afterwards, the weighed mass of CP was soaked in 100 mL of 0.05% *w*/*v* of propyl paraben which was left for 24 h to form swollen mass. To eliminate CP particle clumping [13], the swelled form was stirred at 1000 rpm for 10 min using a mini basic lab scale Qiangzhong^®^ (Shenzhen, China) homogenizer [14]. In formulations containing polymeric blend of AG-CP (F9–F12), the previously prepared respective amount of gels of AG and CP were homogenized for at least 10 min or till no clumping was observed (Table 1). In all prepared formulations, 0.35 mL of DMSO containing the dissolved amount of drugs were added. Finally, 0.15 mL glycerol was added into this dispersion mixture and was then mixed further for 5 min. The pH of the prepared formulations was set in the range of 6.7–7.0 with triethanolamine [15]. Alternatively, drugs were not added into gels when subjected for mucoadhesive evaluation in healthy volunteers.

### 2.4. Solid State Studies

#### 2.4.1. Fourier Transform Infrared (FTIR)

FTIR analysis was performed on the pure samples of TIB, BZN, CP, AG, and of the physical mixture as used in the optimized formulation. Concisely, infrared spectra in the range of 4000–400 cm^−1^ were obtained by operating a Bruker (Billerica, MA, USA™) spectrophotometer (run by OPUS^®^, Alpharetta, GA, USA) using platinum-ATR with four scans in the transmission mode [7].

#### 2.4.2. Differential Scanning Calorimetry (DSC)

DSC analysis was performed on pure drugs and polymers, as well as their physical mixture as used in the optimized formulation. For this purpose, each sample weighing approximately 10 mg was placed in aluminum pan sealed with lids in a Modulated DSC TL^TM^ Q2000 (TA Instruments, New Castle, DE, USA) machine operated by Universal Analysis Software. The response of the thermogram was obtained at a rate of 20 °C/min over a scanning range of 40 to 250 °C using nitrogen as an inert purging gas at a rate of 50 mL/min [16].

#### 2.4.3. X-ray Powder Diffraction (PXRD)

The samples of pure drugs and physical mixture of drug- polymers were evaluated for PXRD analysis by exposing to MiniFlex^®^ (Ventura, CA, USA) 600X-ray diffractometer (Rigaku^®^, Tokyo, Japan) to observe changes in their physical forms. To accomplish this, the samples were subjected to incrementing voltage of 40 kV with current in the range of 15 mA by keeping exposed angle (2 theta) in the range of 5–45° [17].

### 2.5. Physicochemical Characterization

#### 2.5.1. General Appearance

The formulated sustained release mucoadhesive buccal gels were examined for their clarity, syringibility, and presence of any insoluble or precipitated particles and color [18].

#### 2.5.2. pH

The electrode of the pH meter was protruded approximately 3 mm deep inside the formulated gels already placed in the petri dishes and the average reading of three values were taken after the fluctuation stopped [19].

#### 2.5.3. Spreadibility (SP)

Concisely, 0.5 g of the mucoadhesive gel formulation was placed on a pre-marked 1 cm diameter (D1) circle on a clean and dry petri dish. Care must be taken that it should not move outside the circle. Then, a weight of 500 g was added in such a way that the gel was sandwiched between the two petri dishes where weight was imposed from the top of petri dish. The gel attained a new position after spreading (D2). The average of three SP experimentation of each gel formulation was assessed according to the Equation (1) given below [20].

(1) $$\mathrm{Spreadibility}\;\left(\%\right)=\frac{D2}{D1}\times 100$$

#### 2.5.4. Contents Uniformity

The amount of buccal gels equivalent to two loaded doses of BZN and TIB were taken and placed in a clean and dry pestle and mortar. The polymeric structure of gels was completely destroyed by triturating with ample volume of dissolution fluid (0.25% *w*/*v* SLS solution adjusted to pH 6.8). It was then finally diluted to 900 mL with dissolution fluidfollowed by spinning around 800–1000 rpm for approximately 45 min at 37.5 °C. Afterwards, 5 mL of the fluid was taken and filtered using a Millipore^®^ syringe and run on a HPLC machine for the estimating contents of BZN and TIB [15]. The HPLC instrumental settings have been detailed under in vitro drug release. The procedure was repeated thrice.

#### 2.5.5. Swelling Index (SI)

Precisely weighed (W1) of each formulated gel was placed on a glass slide which was then immersed in separate petri dishes each containing approximately 10 mL of pH 6.8 phosphate buffer solution (PBS) maintained at 37.5 °C. For observation, the glass slide was removed from respective petri dish and was weighed to evaluate SI at define time points. Extent of imbibition (W2) was expressed as SI for specific time value using the Equation (2) [21].

(2) $$\mathrm{SI}=\frac{W2-W1}{W1}\times 100$$

#### 2.5.6. Matrix Erosion (ME)

The swelled formulations were placed in a hot oven at 60 °C till the weight remained constant for 24 h. The weight loss during storage in the oven caused by exposure to temperature was evaluated by weighing (W3) again. The ME (%) was calculated using Equation (3) [15].

(3) $$\mathrm{ME}=\frac{W3-W1}{W1}\times 100$$

#### 2.5.7. Ex Vivo Mucoadhesive Strength (MS)

**Figure 1:**
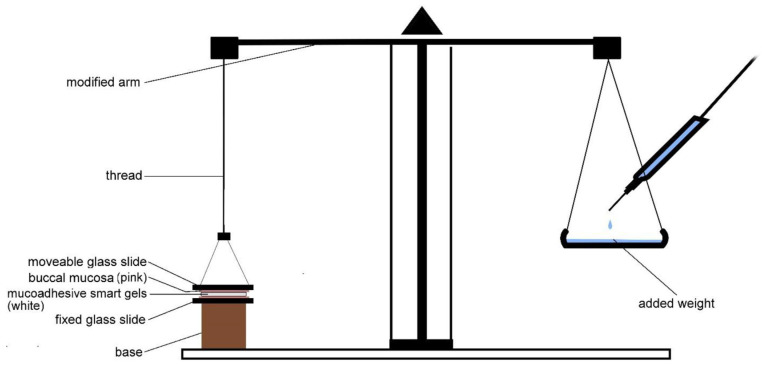
Modified physical balance for the estimation of ex vivo mucoadhesive strength as reported [16].

A modified balance with one arm replaced with base was used for the assessment of MS. For that purpose, the buccal gel was sandwiched between the freshly sacrificed buccal mucosa of a rabbit cheek (Figure 1). The mucosa was glued previously between the glass slides facing the gel. One glass slide was fixed on the base whereas other remained moveable with the thread [16]. After the set up was complete and in static equilibrium, weight was slowly added on the other until the pan detached from the glass slide. The weight required to detach the gel from either of the surface was considered as the respective average MS of the gel after repeating process three times [22].

#### 2.5.8. Ex Vivo Mucoadhesive Time (ET)

**Figure 2:**
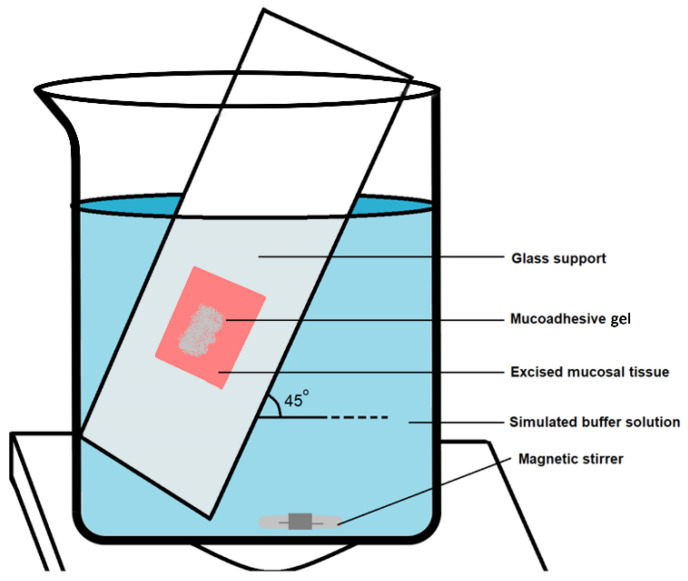
Experimental set up for the determination of ex vivo mucoadhesive time of buccal gel formulations.

Experimental conditions were set up to estimate the ex vivo mucoadhesive time of buccal gel formulations, as reported in literature [23]. For that, an inclined glass slide at an approximate angle of 45° was placed in a beaker upon which a freshly excised rabbit’s buccal mucosa was fixed with the help of acrylate adhesive (Figure 2). The whole set up was immersed in a PBS maintained to pH 6.8 with a volume of 800 mL. Before immersing the slide, 3 g of mucoadhesive buccal gel was applied on an area of 4 cm^2^ to the mucosal surface and was left undisturbed for almost 20 s to develop mucoadhesion. The slide was then immersed gently and the recorded time for which the gel either eroded or detached from the mucosal surface was ranked as ET for each respective formulation [16]. The experiment was repeated thrice.

#### 2.5.9. Ex Vivo Mucoadhesive Flow Time (FT)

**Figure 3:**
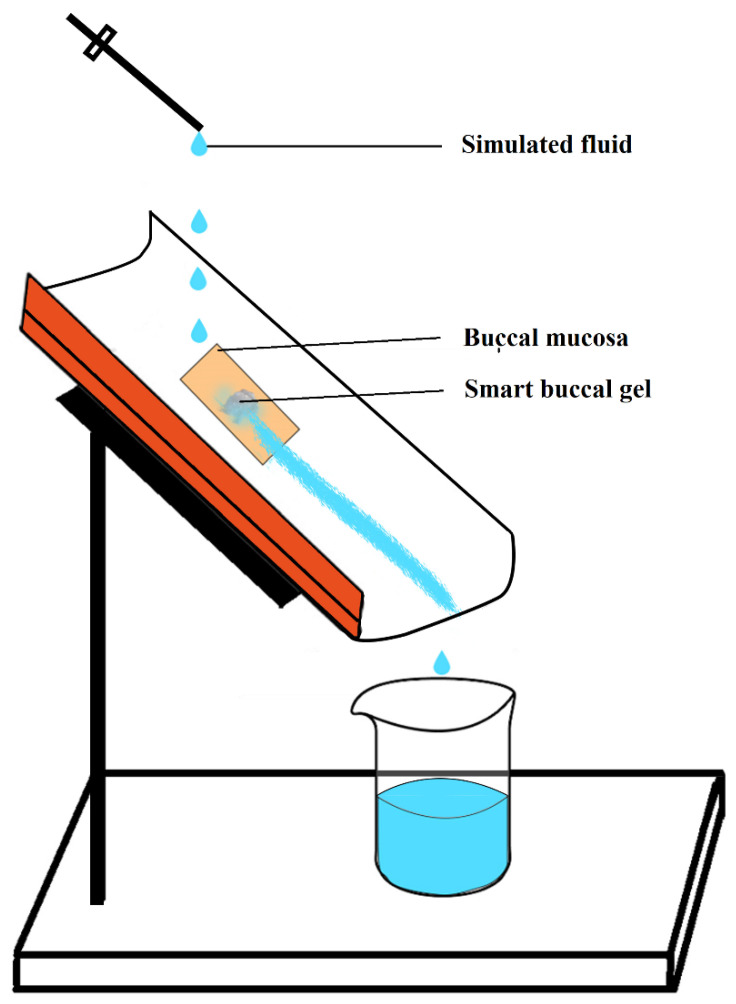
Simulation for estimation of ex vivo mucoadhesive flow time as reported [24].

The FT for the prepared buccal gels was also evaluated using a modified apparatus [24]. Briefly, half of the polyvinyl chloride pipe was inclined at an approximate angle of 60° on which a freshly excised rabbit’s buccal mucosa was adhered using acrylate gum. Afterwards, mucoadhesive smart gel was placed on the surface of mucosa and was kept undisturbed for 20 s to develop mucoadhesion (Figure 3). From the above side of the inclined setup, a consistent flow rate of 1 mL/min of PBS adjusted to pH 6.8 was added drop wise on the gels. The temperature of PBS was kept at 37.5 °C. The time in which the gel completely wiped off the mucosal surface was considered as the average FT for each respective formulation when the procedure was repeated thrice [15].

#### 2.5.10. Ex Vivo Mucoadhesive Drag Force (DF)

**Figure 4:**
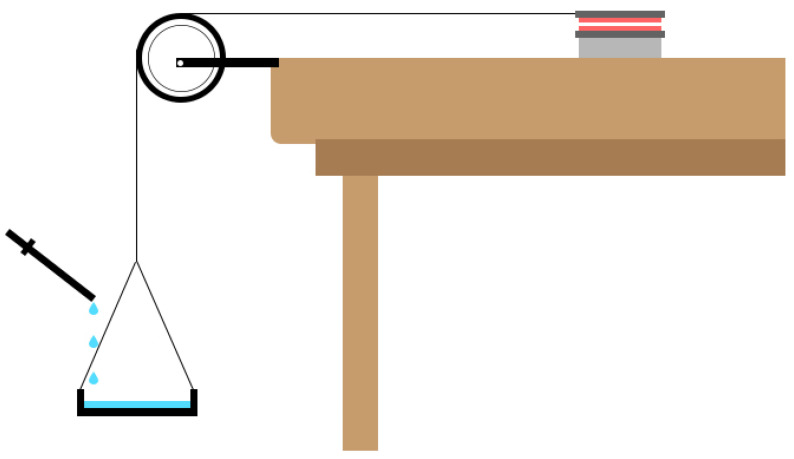
Simulation of estimation of ex vivo mucoadhesive drag force.

The dragging force was observed after the dosage form was sandwiched between the moveable and immovable layer of glass slide containing rabbit’s buccal mucosa on its surface (Figure 4). Concisely, a freshly procured buccal mucosa was obtained and glued to the glass slide surface in such a way that it sandwiched the gel scaffold. Gentle force was applied for 10 s to ensure placement of gel between the mucosal layers. The moving glass slide was attached with the thread so that it could allow the detachment along the horizontal direction upon the addition of the weight as shown in the Figure 4. The weight after which either of the mucosal slides was detached or travelled a distance of 1 cm on the buccal mucosa was considered as the DF of each respective gel scaffold. The procedure was repeated three times to obtain an average value.

#### 2.5.11. Mucoadhesive Time in Healthy Volunteers (MT)

Concisely, drug free formulated buccal gel was gently applied in between the lower gum and the inferior labial frenulum of the healthy volunteers. The volunteers of either gender, aged between 20 and 27 year, not suffering from any acute disease nor xerostomia and who were willing to participate in the study, were included. The volunteers were advised not to intake solid food during the entire experiment but were not prohibited for liquid diet. Also, the rinsing of buccal cavity was not allowed [25]. The volunteers were guided to record observations during the time period. Periodic inspection with respect to the condition of gels in the cavity was also evaluated. The time at which gel either broke into pieces or eroded from the point of application was considered as the in vivo MT of volunteers and was obtained as the standard deviation of average value [26].

#### 2.5.12. In Vitro Drugs Release

The simultaneous in vitro drugs release studies of both drugs were carried out using the simulated conditions to mimic pH of buccal salivary. To accomplish, 3 g of the gel equivalent to double doses of BZN and TIB were placed in a watch glass. It was enclosed from the top with a 100 mm mesh [15]. The watch glass was then immersed in a USP dissolution vessel or a beaker containing 900 mL of 0.25% *w*/*v* SLS solution adjusted to pH 6.8 [17]. The dissolution medium was already maintained at 37.5 ± 1 °C with a rotation speed of 50 rpm throughout the experiment.

#### 2.5.13. HPLC Instrumental Settings

Samples of 5 mL were withdrawn from the media and replenished with fresh dissolution volume. After filtration, it was run on a Agilent^®^ (Santa Clara, CA, USA) HPLC 1260 Infinity machine to quantify drug release by analyzing each sample thrice [27]. The mobile phase was composed of acetonitrile and potassium dihydrogen phosphate in a volumetric proportion of 7:3 (*v*/*v*). The pH of the mobile phase was adjusted to 4.5 with o-phosphoric acid. The temperature of the C_18_ column was maintained at 35 °C with a flow rate 1 mL/min of mobile phase. The retention time of benzocaine and tibezonium iodide were 2.29 and 4.15 min, correspondingly.

#### 2.5.14. In Vitro Drugs Release Kinetics

The mode of drugs release from the optimized buccal gel was studied by applying different pharmacokinetic models such as zero order, first order, Higuchi, Korsmeyer-Peppas and Hixson-Crowell models using DD solver^®^ software. The model depicting the highest value of coefficient of regression was considered as the best fit model for drug release [28].

#### 2.5.15. In Vivo Volunteer Adaptability Response

The in vivo adaptability response of the volunteers towards formulated gels was evaluated. Included parameters were dryness to mucosa, inflammation in the buccal cavity, pain or irritation, dislocation of the gel from initial point of adhesion [17]. Clinical observations were estimated during the evaluation of mucoadhesive time in healthy volunteers. The findings were then compared with smart mucoadhesive buccal gels composites where low concentrations of polymers were used to formulate gel [15]. The formulation was tested on five healthy volunteers.

#### 2.5.16. In Vivo Salivary Drug Concentration

The salivary drug concentration from the optimized formulation was assessed on three healthy human volunteers. For that purpose, volunteers of either gender aged between 18 and 27 years were assured not to consume any food during the time interval and allowed to drink water 15 min before each sampling time. However, liquid form of food was allowed during the experiment [25]. At pre-defined time intervals, approximately 200 µL of sample was obtained from the frontal portion below the tongue and diluted to 2 mL with 0.25% *w*/*v* SLS adjusted to pH 6.8 [15]. Then, around 1 mL of acetonitrile was added to precipitate any mucilaginous form in saliva. Afterwards, the sample was placed in a small petri dish at 50 °C to remove the organic layer followed by centrifugation at 8000 rpm at 25 °C and finally the supernatant was collected by filtering thorough a Millipore syringe filter of 0.2 µm. The obtained filtrate was then eluted into a Agilent^®^ HPLC 1260 machine with auto sampling vials to estimate the quantitative salivary drug release [27]. From the salivary release data, different pharmacokinetic parameters such as maximum drug concentration (C_max_), time (t_max_), area under the curve (AUC_0–t_) and extrapolated area under the curve (AUC_t–∞_) were calculated. The procedure was repeated on five volunteers.

#### 2.5.17. Stability Study

The stability studies were performed on the optimized mucoadhesive buccal sustained release gel at 40 °C with relative humidity (RH) of 70% ± 5 and up to 6 months [29]. In each interval, the buccal gel was observed for parameters such as physical appearance, drug contents mucoadhesive strength (MS), mucoadhesive drag force (DF), mucoadhesive flow time (FT) and contents uniformity. The gels were placed in an air tight glass test tubes and sealed with plastic lid and adhesive tape [30]. The in vitro drugs release after exposure to stability conditions was also performed to estimate the similarity of release patterns affected by stability using similarity (*f*_2_) and dissimilarity (*f*_1_) factor tools [31]. In addition, sample paired *t*-test was applied to confirm whether significant difference between the release of drugs existed or not during exposure to stability conditions [22].

## 3. Results and Discussion

In the preliminary phase, AG was evaluated for mucoadhesive properties as the compressed dosage form [7]. However, it possessed poor force and even the concentration (% *w*/*w*) of AG was increased. Therefore, the potential of AG in mucoadhesive gel form was evaluated. In some unpresented trial batches, the concentration of AG was optimized based on drug release (data not shown). However, the concentrations of both polymers were then set for prolonged release.

### 3.1. Solid State Characterization

Solid state studies such as FTIR, DSC, and PXRD in the physical mixture of the optimized polymers and of pure drugs were performed whereas stability studies were only conducted on the optimized formulation.

#### 3.1.1. FTIR Spectral Analysis

**Figure 5:**
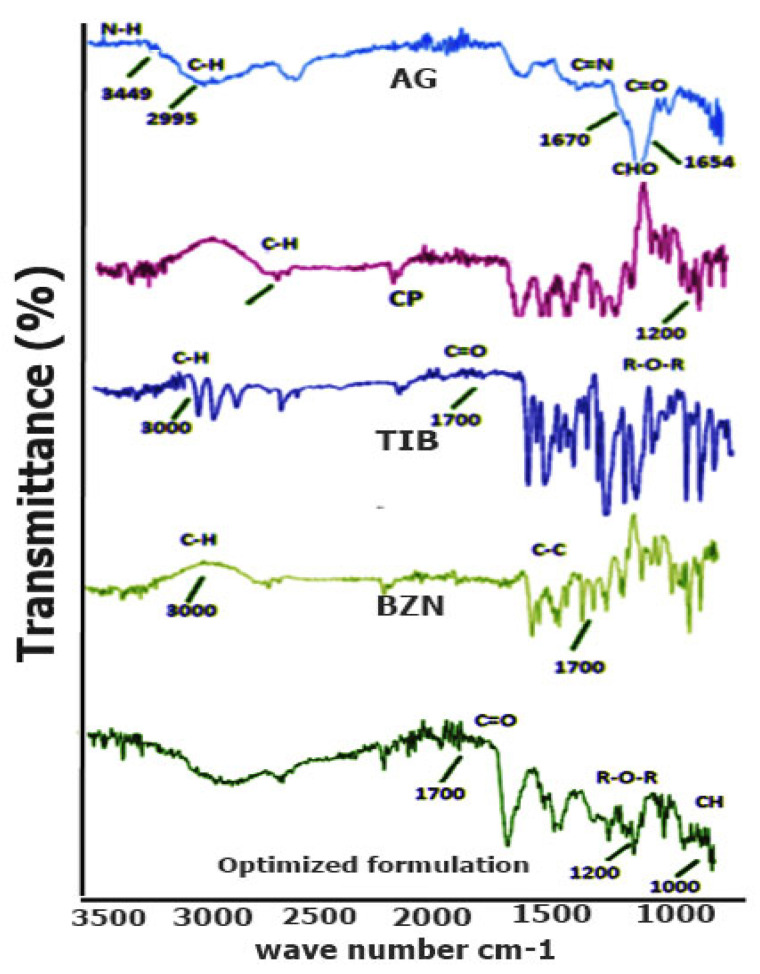
FTIR of AG, CP, TIB, BZN and of the physical mixture according to optimized formulation.

Owing to the specific energy absorption by functional groups in the molecule, FTIR interpretation is a simple procedure to identify the specific absorption regions. The FTIR spectral analysis on CP depicted prominent stretching vibration of the carbonyl group (C=O) between 1750 and 1700 cm^−1^, whereas the peak in the region of 1450–1400 cm^−1^ showed the C-O or O-H stretching of the molecule [32] as seen in Figure 5. The band spectrum ~1250- 1200 reflected the C-O-C of the acrylate derivative [29]. The R-O-R band was shown by the peak around 1164 cm^−1^ indicating its stretching vibration. The peak between the 850 and 800 cm^−1^ represented the C-H out of the plane bending for carbomers [33]. In the spectrum of AG, the band at 1646 cm^−1^ corresponded to the bending of O-H group in the polymer. The specific absorption band of the polymer as well as the C-H bending vibrations of the anomeric carbon existed ~928 cm^−1^ and 889 cm^−1^, respectively [34]. In the optimized formulation (T2), a minor shift in the peaks predicted strong absorption of C-H bending vibration band to 898 cm^−1^. The reason for this was that hydrogen bonding between the polymers caused the band to shift to the newer value.

#### 3.1.2. DSC Analysis

**Figure 6:**
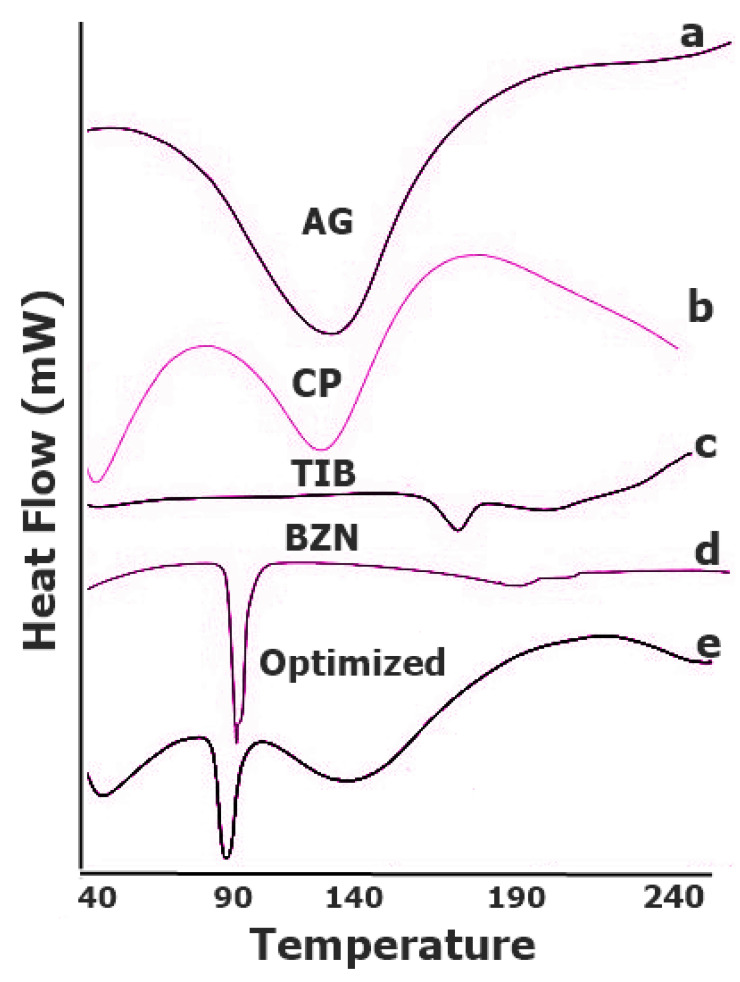
DSC peaks of (**a**) AG, (**b**) CP, (**c**) TIB, (**d**) BZN, and (**e**) physical mixture.

In order to detect any sort of interaction among the components of the formulations, DSC analysis was performed on pure drugs, polymers, and their physical mixture (Figure 6). As depicted, the melting point of BZN drug was identified by an endothermic event corresponded to the temperature of 89.52 °C. The powder of the drug started melting at this temperature but a sharp depression was achieved at 92.15 °C, which is similar to the reported work [35]. For TIB, melting of the pure drug was confirmed as an endothermic peak depression around 161.40 °C which also corresponded to recent findings [17]. For Carbopol, the characteristic endothermic peak was found to be similar as reported in the literature [36,37]. Similarly, the pure AG started melting around 88–93 °C, depicting the amorphous nature of the polymer [38]. No additional or unusual peaks were found in the DSC of optimized formulation (T2). While the sharp endothermic event observed in the optimized formulation approximately around 90 °C was associated with the BZN drug. This showed that the integrity of the drug was maintained in the mucoadhesive dosage form. However, the enthalpy of the heat value was reduced for BZN.

#### 3.1.3. PXRD Analysis

**Figure 7:**
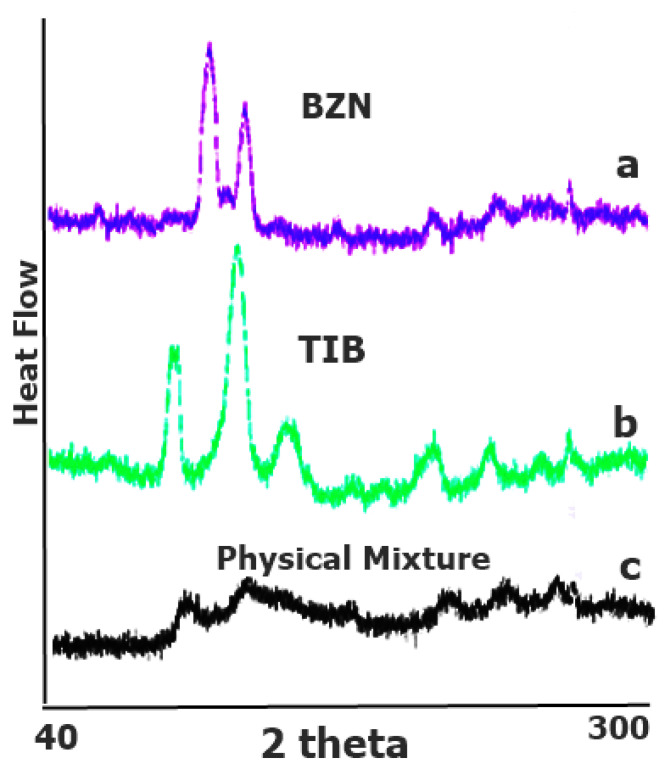
PXRD pattern of (**a**) BZN, (**b**) TIB, and (**c**) the physical mixture.

The PXRD results of BZN and TIB revealed crystallographic patterns with sharp narrow characteristic peaks (Figure 7a,b) [35]. This cluster pattern was distinguishable for drugs in pure form and is indicative of crystal pattern [39]. However, the PXRD pattern of the optimized formulation clearly revealed reduction in intensity of incorporated drugs in the physical mixture (Figure 7c), [40]. This could be linked to less concentration of drugs in physical mixture due to which the intense signals were reduced. This can be seen with small intensity peaks in the PXRD of pure drugs, which suggests that the physical form of the drugs was not changed in the formulation.

### 3.2. Physicochemical Evaluation of Buccal Gels

The physicochemical evaluation of sustained release smart mucoadhesive buccal gels is as follows:

#### 3.2.1. General Appearance

As compared with outcome of smart mucoadhesive buccal gels composites, the appearance of AG based gels (F1–F4) was similar and transparent. In contrast, the translucency of CP based gels (F5–F8) was increased as the function of increased concentration of CP. Linked to the results of smart mucoadhesive buccal gels composites, no precipitation or grittiness was observed in the formulations of either type. Similarly, no unusual change in color was observed when the ingredients were mixed during preparation. The findings were supported to similar work on non-monotonic swelling of agarose and carbopol interpenetrating gel scaffold. In terms of consistency, the AG gels (F1–F4) were more stiff in appearance [41] while the (F9–F12) appeared to have a gelatinous morphology [42]. The composite gels (CG1–CG4) revealed consistency that was more like AG gels which clearly reflected that AG predominantly affected the consistency of gels. All of the formulated gels were non-syringable with the ordinary suction force of 25G plunger.

#### 3.2.2. pH

The pH of all formulations was found to be in the range of 6.85–6.97. As the pH of formulated gels was adjusted with triethanolamine, it was designed to maintain near to neutral pH value. It was found that agarose showed better swelling phenomena near to this value [13]. Studies supported that when the pH of the gel system was near to 7.0 value, the swelling became better due to the viscous and strong percolated networks of CP gel [43].

#### 3.2.3. Spreadibility (SP)

Certainly, SP has a role in maintaining the interaction of mucus with buccal gel due to its spreading along the surface area of mucosa caused by squeezing pressure between the cheek and gingiva. Irrespective of the polymer, the lower values of polymeric concentration in gel is generally associated with better spreadibility. Hence, as the concentration is increased, the interaction between the polymer and hydrophilic forces in water increases resulting in more viscous gel and less spreadibility. The same scenario was observed with mucoadhesive buccal gel formulations in the sustained release gel scaffold study. A higher SP value was associated with CP containing formulations (F5–F8) as compared to AG or their polymeric composites. The general order of SP was SC > GC > SA. The highest value was observed when CP was formulated in the lowest concentration such as 0.5% *w*/*v* (F5) and then decreased gradually as function of increasing polymer concentration. Intensity of SP index was lowered when gels were formulated with AG alone and the lowest value of 75.7%was observed in 1.25% *w*/*v* concentration (F4). This spreading phenomenon was based on polymeric concentration since the values were poor compared to the gel formulated in smart mucoadhesive buccal gel composites (Table 2). While in CP polymeric gel composites F5-F8, the SP was greater as compared to F1–F4 containing AG only. It can be stated that formulations with CP alone possessed better SP and the polymer gel composite of AG-CP improved the swelling properties of AG.

#### 3.2.4. Contents Uniformity

For stability as well as proper drug release from the delivery system, the contents of the dosage form must be uniform in terms of its identity and integrity. The content uniformity of formulated buccal gels was evaluated based on quantitative estimation of both drugs. The procedure was performed according to the developed HPLC analytical procedure. The results showed the contents of BZN were in the range of 98.62–103.19%, whereas TIB contents ranged between 95.51 and 103.73%. The value showed fair uniformity of drugs in the formulated gels.

#### 3.2.5. Swelling Index (SI)

**Figure 8:**
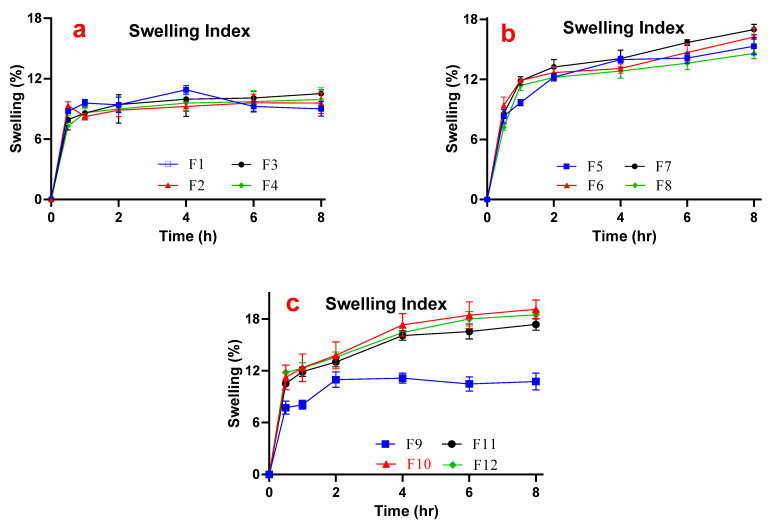
Swelling trend of formulations containing (**a**) AG alone (F1–F4), (**b**) Carbopol alone (F5–F8), and (**c**) AG-CP composite (F9–F12).

Swelling is a property that can be linked with the release of a drug [44]. This is due to the fact that the extent of influx of water for definite swelling will also govern the rate at which the drugs will efflux from dosage form, showing that the extent of drug release can be adjusted depending upon the nature and concentration of swellable polymer. In the sustained release gel scaffold study, the gel swellability pattern of formulated gels was almost similar to smart mucoadhesive buccal gels composites and an overall increasing trend was observed for all of the gel formulations. In contrary to smart mucoadhesive buccal gels composites study, the magnitude of swellability was not proportionally increased when the corresponding concentration of polymers in gel was increased. This meant that there was a definite swelling capacity for polymers, after which the swelling was not further increased even when the concentration of polymer was raised. In gels containing AG alone, maximum SI of 10.89% after 4 h was observed for formulation F1. It can also be observed from Figure 8a that formulations F1–F4 swelled to a pre-equilibrium level after 0.5 h. The findings are supported from agarose-carbopol based hydrogels synthesis in a study for tissue engineering [45]. After a sharp initial swelling at 1 h, the formulations swelled very slightly till 8 h [46]. Afterwards, the formulations did not exhibit a steep rise in swellability over time rather just a slow increasing value was seen compared to F5-F8 formulations. It can also be observed that swelling was better when AG alone was formulated in F1 (0.5% *w*/*v*) and F2 (0.75% *w*/*v*) as compared to F3 and F4.

In CP alone formulations (F5–F8), there was a general steady increase in the SI values based on the concentration of CP in gels and the swellability reached to its maximum values till 8 h. Maximum SI of 16.98% was observed with F7 till 8 h. However, the swellability was improved when the polymeric gel composite was formulated. The maximum SI rose to 19.12% (8 h) in formulation SG2 containing 0.75% *w*/*v* AG and CP each, the second highest of 18.48% by SG4. Opposite to individual polymeric gels, the swellability of polymer composite gels was lowered when the gels were formulated with lower concentration of the polymers as noticed in SG1 (Figure 8c). Overall, it reflects that when AG and CP were combined, the swellability was improved than either alone. The findings were supported by better swelling of AG and CP gel composites [13]. In terms of swellability, the trend of polymer composite gels (Figure 8c) was identical to that of steep trend depicted by CP (Figure 8b).

#### 3.2.6. Matrix Erosion (ME)

Matrix erosion (ME) is an estimation that what extent of the buccal gel can degrade through fragmentation or erosion. The value of ME was higher > 85% but still lesser than the gels formulated in smart mucoadhesive buccal gels composites study probably due to lower concentration of polymer. However, there was not a defined pattern observed for ME (Table 2). The lowest values were attained with formulation F2 and F4 containing 0.75 and 1.25% *w*/*v* of AG, respectively, while the highest values were associated with F5, F10 and F12. The higher extent of erosion rate could be linked with the higher amount of water present in the gels.

#### 3.2.7. Ex Vivo Mucoadhesive Strength (MS)

**Figure 9:**
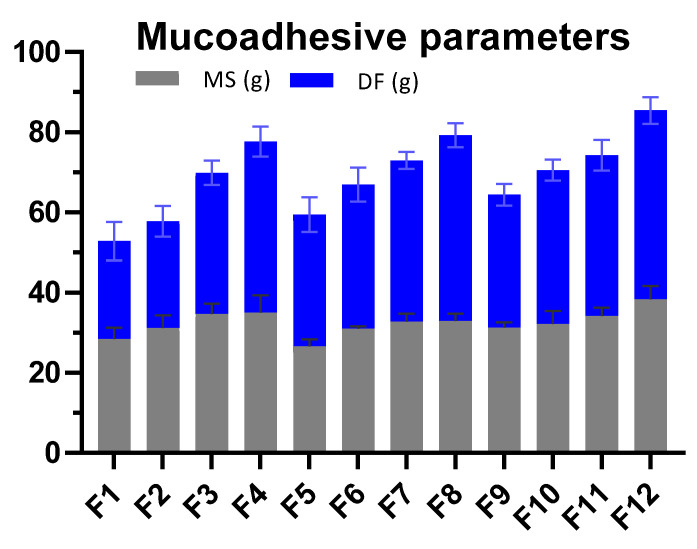
Comparison of the mucoadhesive strength properties of different gel fabricated formulations.

Adhesion to the mucosal membrane is a definite property of mucoadhesive polymers which ensures smooth attachment of the dosage form for designed period [47]. The adhesion found in smart mucoadhesive buccal gels composites supported the mucoadhesion of the prepared formulations ex vivo and in vivo. The MS evaluation on rabbit’s buccal mucosa revealed that an increasing concentration of polymeric gels was associated with increasing MS [16]. In comparing, the MS of AG alone (F1–F4) gels was found superior to formulations containing CP alone (F5–F8). However, the MS of polymer composite gel (F9–F12) was further superior to its respective F1–F4. Highest mucoadhesive strength of 38.35 g was found with F12 (Figure 9) The MS values of respective gels were superior to its respective gel formulations prepared in the smart mucoadhesive buccal gels composites which could possibly be due to concentration-based 3D swelling of gel polymer composite thus enhancing its adhesion [13].

#### 3.2.8. Ex Vivo Mucoadhesive Drag Force (DF)

The DF can be considered as a parameter to measure how much force is required to initiate movement after the gel is applied on the buccal mucosa which provides a rough estimation of the in vivo performance of gels. The results of DF were somewhat similar to MS since both parameters were linked to adhesive force. The value of DF was increased as the concentration of AG, CP alone or its composite was enhanced in gel formulation. The maximum DF of 47.07 g was found for F12 (Figure 9). The results exhibited that polymeric gel composite possessed better mucoadhesive drag force than either polymers alone.

#### 3.2.9. Ex Vivo Mucoadhesive Time (ET)

**Figure 10:**
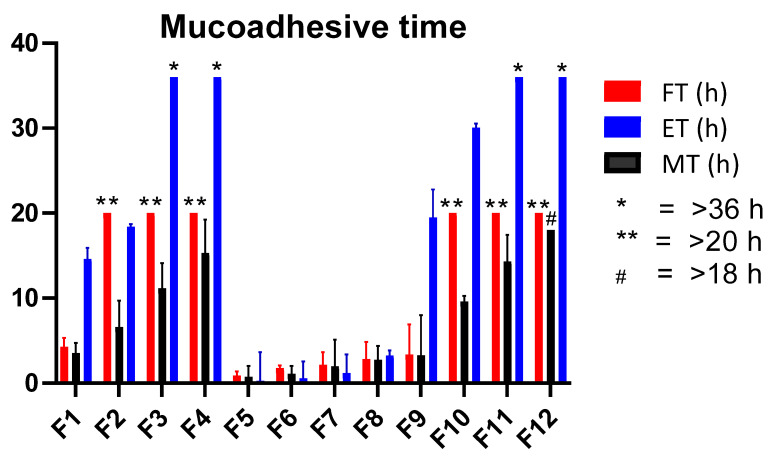
Comparison of the different mucoadhesive time properties of fabricated scaffold gel.

The ex vivo mucoadhesive time is an estimation of how long the mucoadhesive buccal gels can withstand the dissolution fluid. A low value indicates poor mucoadhesion and there could be a possibility that the dosage form may detach from the buccal mucosal surface in its intact form. The results from the experimental values of ET revealed a significant difference. The values for formulations containing CP alone (F5–F8) were quite less than AG alone and AG-CP combined formulations. This is mainly attributed to the adhering capability of AG to biological macromolecules [48]. Under simulated ex vivo conditions, the formulations F5–F8 exhibited a mean time of less than 4 h (Figure 10) while rest of the formulations provided a mucoadhesion time of more than 14 h. Formulations containing AG ≥ 1.0% *w*/*v* exhibited a durable adhesion to buccal mucosa which was greater than 30 h. Since the strength of AG of agarose increases with increase in pH, as a result, its swellability is maximum near to neutral pH. A study reported that the superior mechanical property and stiffness of agar gels is due to the anhydrous backbone which is particularly present in disaccharide units of agarobiose. Since, AG is composed of repeating units of anhydrous agaropyranose in hydrogels form, so it can be assumed that the adhesive property of AG is attributed through the anhydro units [49]. Furthermore, during the annealing of AG hydrogels, the polymers are cross-linked to produce effective strength. This whole scenario results in better mucoadhesive strength and time of formulated gels. It was found that the ET of polymer composite gels (F9-F12) was superior compared to the individual gels of either AG or CP.

#### 3.2.10. Ex Vivo Mucoadhesive Flow Time (FT)

A significant FT buccal formulation is critical since lower values of time may cause dislocation of the dosage form due to the drooling action of saliva. In fabricated gels, higher values of FT were found with composite gels (F9–F12) as a function of increased concentration of composite polymers (Figure 10) as compared to AG or CP alone gels. This indicates the interpenetration of both polymers to possess better FT [13]. Highest FT response of 18 h was observed when AG and CP were fabricated in F12.

#### 3.2.11. Mucoadhesive Time in Healthy Volunteers (MT)

The MT is a predictor character of gels showing their behavior in the buccal cavity. Volunteers were allowed to drink liquid during the experiment. In volunteers, the CP based F5–F8 exhibited a weaker response of MT which can be correlated to the concentration based viscosity of CP gels [50]. The lowest value of time in volunteers was observed with F5 (0.76 h) containing 0.5% *w*/*v* of CP. By further increasing CP concentration did not significantly increase the MT as was observed with AG containing formulations. The agarose or its composite gels exhibited a higher MT in volunteers where highest value was observed with F12 which was still present in the buccal cavity of volunteers at 18 h. The volunteers reported that AG containing formulations primarily did not erode rather slightly “dissolved” in the cavity. There, AG containing formulation also did not disintegrate in contrast to CP [51]. More than 10 h of MT were depicted when AG or its composite was delivered at a concentration of 1% *w*/*v* or above (Figure 10). Converse to the results of smart mucoadhesive buccal gels composites, the MT of gels were less than its respective FT values. It could be due to the reason that at concentrations of 0.5% *w*/*v* or higher, AG formed a stiffed gel [41] and under simulated conditions the gels might adhere to the excised mucosa as a stiffed gel and thus not disintegrate or erode [52].

**Figure 11:**
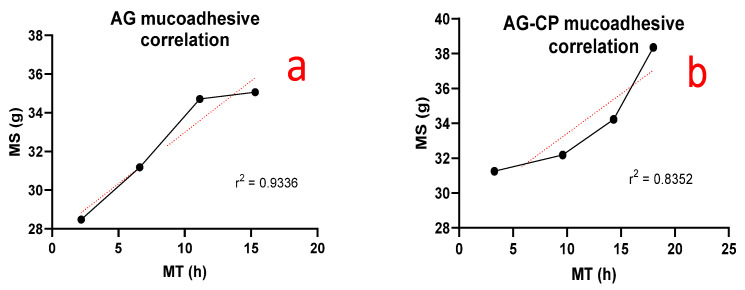
Correlation between ex vivo mucoadhesive strength and mucoadhesive time in healthy volunteers in (**a**) AG based gels (F1–F4) and (**b**) AG-CP based gels (F9–F12).

Correlation between MS and MT can be observed for different concentration of AG alone or its composite form of AG-CP (Figure 11b). It can be clearly observed that the mucoadhesive property of gels in AG-CP increased as the concentration of polymers was increased. While with AG alone (Figure 11a), it showed an increasing correlation of mucoadhesion up to a certain limit. After that behavior in MS was lowered at concentration of gels prepared in the current study (Figure 11a).

#### 3.2.12. In Vitro Drugs Release

**Figure 12:**
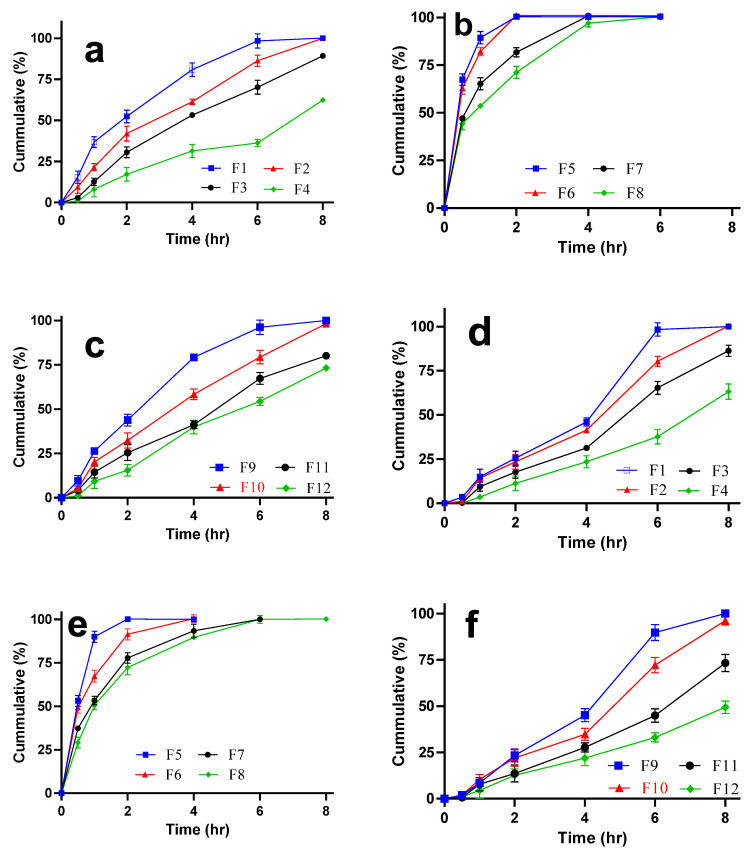
In vitro release of BZN (**a**–**c**) and TIB (**d**–**f**).

The dissolution studies depicted a sustained release of drugs in some formulations (Figure 12a,c,d,f) while formulations containing CP alone (Figure 12b,e) showed drug release up to 6 h. Such formulations containing concentration of CP less than 1% *w*/*v* released both drugs in less than 4 h which depicts the incapability of CP alone to sustained release in gel form. In contrast, formulations containing AG or its composite exhibited a concentration dependent sustain release of both drugs. The sustained release of both drugs in AG based formulations could probably be attributed due to the gel scaffold in which swelling of the formulation was not more than 25% of applied weight. This influx of fluid might have allowed slower diffusion of drug out of gel composites [52]. Also, the release of BZN (Figure 12a) as well as TIB (Figure 12d) became slower as the concentration of AG increased. The release of TIB was incomplete and slowest when it was delivered in a gel concentration of 1% *w*/*v* or above (Figure 12d,f). Moreover, the initial release of TIB was slower as compared to BZN which could be attributed to the solubility reasons. Moreover, the in vitro results revealed complete drugs release till 8 h from F1, F2, F9, and F10 formulations. Also, when the mucoadhesive properties of the formulations were compared, the values of composite gels F9 and F10 were observed superior. Further, when the values of F9 and F10 were analyzed, F10 was considered superior to F9 in showing mucoadhesion and produced more sustained release of both drugs over time. Moreover, the SI of F10 was better than F9 which deduced F10 be the optimized formulation.

Hence, F10 was further evaluated for stability study, in vivo adaptability response in healthy volunteers, salivary drug concentration, in vitro salivary release kinetics, and pharmacokinetic estimation of drugs in healthy volunteers.

#### 3.2.13. In Vitro Drugs Release Kinetics

Kinetic analysis was evaluated on the in vitro and salivary drugs release data of the optimized formulation. In all cases, the best fit model exhibiting maximum value of coefficient of regression (r^2^) was Korsmeyer-Peppas model (Table 3). It depicted the release of drugs from polymeric system of gel composites. For in vitro release of TIB, the value of ‘n’ was 1.212 which suggests super case type II efflux of drug from the gel scaffold [34]. It involves some complex involvement of mechanism of kinetics of drug molecules including diffusion and erosion. The performance of TIB inside the buccal cavity of volunteers displayed release based on diffusion plus erosion of the gel scaffold as the value of ‘n’ was 0.45 > *n* > 0.89. For BZN, both in vitro and salivary release followed diffusion as well as erosion in order to release the drug locally since the value is 0.45 and 0.9 [53].

#### 3.2.14. In Vivo Volunteer Adaptability Response

The response of optimized formulation (F10) in healthy volunteers did not induce any symptoms of inflammation, irritation or pain as long it was attached to buccal mucosal surface. Nor it induced any pathological symptoms of acute oral mucositis post-removal 24 h which shows that mucoadhesive buccal gels were well tolerated by individuals. Similarly, around 6.67% of the participants responded with respect to taste alterations. About 2 participants also responded for moderate salivary increased during the residence of dosage form in the buccal cavity (Table 4). Since the drugs are designed to release as well as act locally, it should increase the salivary excretion to acute increasing level. It would in that case be rinsing the drug from the point of action down into the intestinal tract.

#### 3.2.15. In Vivo Salivary Drug Concentration

The salivary estimation of BZN as well as TIB was evaluated until 10 h to detect the quantitative presence of drug in volunteer’s saliva. For this, the volunteers were evaluated for salivary samples with protocols and procedures that have already been explained under methodology section. The outcome revealed that both drugs initially produced a higher salivary number of drugs. However, the concentration of BZN remained higher than TIB throughout the sampling time points. The drooling action of saliva down to the intestine could be one of the reasons why full drug release was not achieved during that time period. The maximum amount of TIB was 52.18% at 8 h, while in the case of BZN, 59.87% drug was detected in the saliva till 2 h. If the in vitro and salivary correlation of both drugs was examined, it was found that the salivary and in vitro simulated drug release of TIB had a value of 0.8030 as compared to 0.4136 for BZN. The values of salivary drug concentration revealed that the concentration of BZN exceeded 5 µg/mL. It moved below 5 µg/mL at the initial and last sampling interval. While for TIB, in between 2 and 8 h, the concentration of drug was above 5 µg/mL.

**Figure 13:**
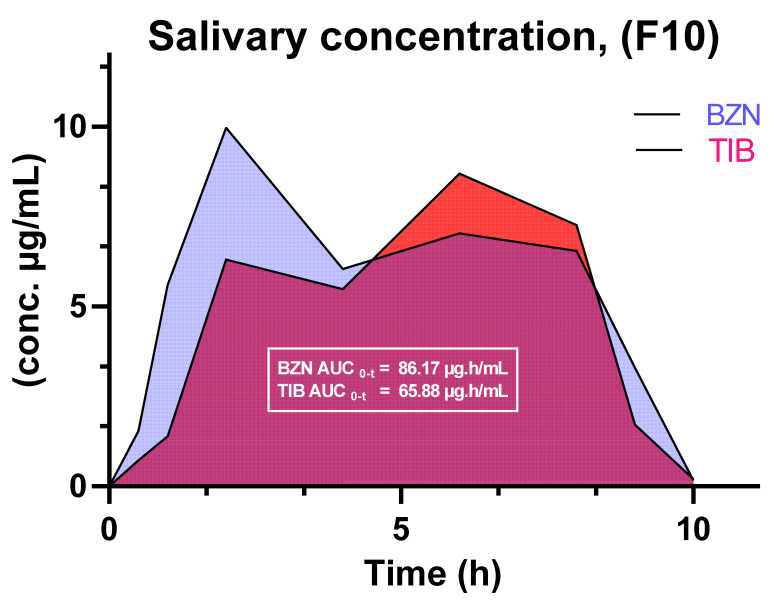
Area under the curve (AUC) for BZN and TIB as a function of time.

Similarly, the pharmacokinetic estimation of drugs in the saliva of human volunteers through non-compartmental approach showed that C_max_ of BZN (9.97 µg/mL) was achieved early at 2 h, whereas the corresponding value of TIB (8.69 µg/mL) was obtained around 6 h. The values were comparatively higher as reported in our previous studies to deliver drug for better salivary concentration [15,17]. The C_max_ of BZN was higher than that of TIB while both drugs showed 2 plateau regions (Figure 13) which could be attributed due to the clearance of drugs by rinsing action of saliva. The elimination rate constant for BZN and TIB were 0.24 and 0.71 h^−1^, respectively. With a dose of 15 mg each for each drug from the optimized formulation F10, the AUC_0-t_ for BZN and TIB were 59.28 and 55.75 µg·h/mL, respectively. When AUC_t–∞_ was assessed for both drugs, it was found that the contribution of BZN was significant from the last time interval to infinity, while not for TIB. So, additional time interval of 10 h was also evaluated for drugs in order to fulfill the significance of AUC from time t_10h_ to infinity. The cumulative AUC _0–∞_ for BZN and TIB were 86.17 and 65.88 µg·h/mL, respectively (Table 5).

#### 3.2.16. Stability Study

**Figure 14:**
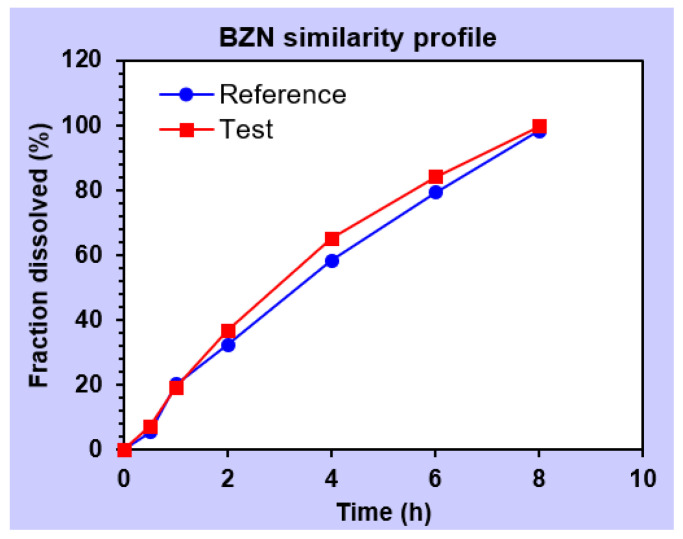
Diagram depicting the similarity profile of BZN before (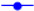) and after (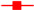) stability conditions.

**Figure 15:**
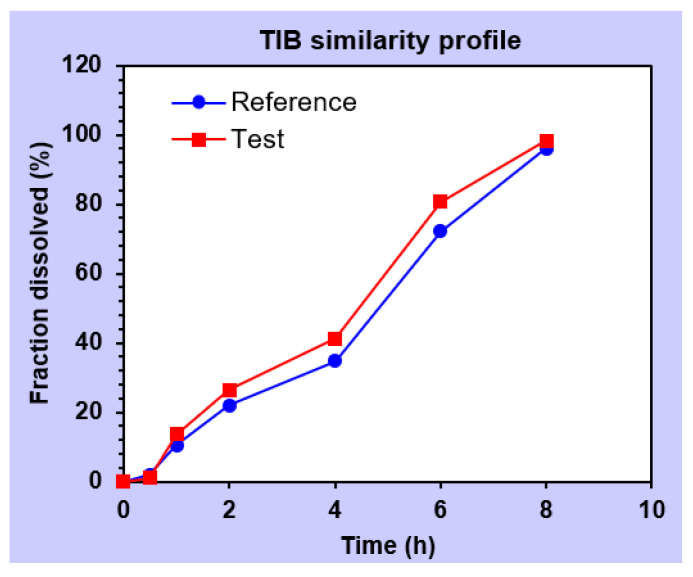
Diagram depicting the similarity profile of TIB before (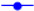) and after (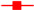) stability conditions.

During storage, the optimized formulation (F10) was evaluated for some physicochemical tests and drug contents, as well as the release of BZN and TIB. It was observed that there was no notable change in physical appearance, which was in accordance with the smart mucoadhesive buccal gels composites. At the end of stated stability time, F10 did not show any signs of precipitation, color change, translucency or separation of water from the gel phase. Parameters such as pH and mucoadhesion was not significantly different from the formulated gels. Dissolution studies (Figure 14 and Figure 15) were also carried out to evaluate significant difference in similarity and dissimilarity factor (Table 6) which depicted that the respective values of similarity and dissimilarity factors were within the acceptable limits [17]. For BZN, the corresponding values of *f*_1_ and *f*_2_ were 7.0 and 70.72 whereas it was 10.79 and 66.19 for TIB, respectively.

Similarly, student’s paired *t*-test was applied on the release data before and after stability which showed *p* value of ≤ 0.05 for both drugs, indicating that no significant different existed between the release profile of BZN and TIB (Table 7). The formulation also retained its total drug contents safe during the stability period which concludes that the optimized formulation (F10) was better to maintain its integrity and physicochemical parameters.

## 4. Conclusions

In pursuit of our research objectives, physical and physicochemical tests were performed on the formulated buccal dosage forms to estimate mucoadhesive capability of agarose (AG). In the sustained release gel scaffold study, AG mainly contributed to the mucoadhesive properties in the sustained release buccal formulations. The mucoadhesive MT, ET, MS, DF, and FT values were more dependent upon the concentration of AG in the formulation compared to CP. The maximum exhibited values by F12 were MT (>18 h), ET (>36 h) and FT (>20 h), MS (38.35 g) and DF (47.07 h). The MS for CP were less when compared with DF converse to AG based gels. The values were poor when CP was formulated alone without AG in formulations (F5–F8). Similar to the findings of smart gels, the optimized dosage form was stable under storage stability conditions and did not produce any adverse response in the buccal cavity of volunteers. The C_max_ value of TIB (8.69 µg/mL) and BZN (9.97 µg/mL) were obtained with the optimized formulation F10 in the saliva of volunteers. It was deduced that AG possessed better mucoadhesive properties in buccal gel form in comparison to the compressed tablet form and did not evoke any adverse clinical response in the buccal cavity of volunteers. Conclusively, agarose has significant mucoadhesive properties and agarose-carbopol composites could be considered as a potential alternative carrier to deliver drugs through mucoadhesive buccal route.

## Figures and Tables

**Table 1:** Concentration of polymers added in the mucoadhesive smart gels.

Codes	F1	F2	F3	F4	F5	F6	F7	F8	F9	F10	F11	F12
AG (% *w*/*v*)	0.5	0.75	1.0	1.25	-	-	-	-	0.5	0.75	1.0	1.25
CP (% *w*/*v*)	-	-	-	-	0.5	0.75	1.0	1.25	0.5	0.75	1.0	1.25

**Table 2:** Spreadibility and matrix erosion of gels scaffolds.

Code	SP (%)	ME (%)
F1	140.2	90.0
F2	101.9	89.9
F3	88.7	91.2
F4	75.7	89.8
F5	165	93.7
F6	159.4	92.3
F7	144.7	91.6
F8	139.3	91.3
F9	156.1	90.9
F10	134.37	93.4
F11	115.2	93.3
F12	95.56	93.7

**Table 3:** In vitro release kinetics of BZN and TIB from optimized (F10) formulation.

	TIB		BZN	
	Coefficient Value (r^2^)			
	In Vitro	Salivary	In Vitro	Salivary
Zero-order (k_0_)	0.8958	0.7755	0.9801	0.2800
First order(k_1st_)	0.8958	0.8509	0.9700	0.0315
Higuchi (k_H_)	0.8034	0.8758	0.9230	0.3777
Korsmeyer-Peppas (k_KP_) (*n*)	0.9892 (1.212)	0.8851 (0.591)	0.9960 (0.811)	0.8337 (0.490)
Hixson Crowell (k_HC_)	0.9255	0.8298	0.9881	0.1154

**Table 4:** Response of human volunteers (*n* = 15) to in vivo adaptability for the optimized mucoadhesive formulation (F10) in current study.

Parameters	Response (%)	Parameters	Response (%)
**1-Mucosal irritation during adhesion**		**5-Numbness post 4 h administration**	
Yes	-	Yes	100
No	100	No	-
**2-Dosage form displacement from point of administration**		**6-Post removal taste disturbance till 10 h**	
		severe	-
Yes	-	slight	6.67
No	100	none	93.33
**3-Saliva production response**		**7-Mucosal soreness**	
moderate	13.3	severe	-
slight	40.0	slight	-
none	46.7	c- none	100
**4-Numbness post 0.5 h administration**		**8-Mucosal dryness**	
Yes	93.33	Yes	-
No	6.67	No	100

**Table 5:** Pharmacokinetic estimation of BZN and TIB in saliva of healthy volunteers.

Parameters	BZN	TIB
Dose (mg)	15	15
C_max_ (µg/mL)	9.97	8.69
t_max_ (h)	2	6
K_el_ (h^−1^)	−0.24	−0.71
AUC_0–t_ (µG·h/mL)	59.28	55.75
AUC_t–∞_ (µg·h/mL)	26.89	10.12
AUC_0–∞_ (µg·h/mL)	86.17	65.88
AUC_t–∞_ (%)	31.21	17.88
Contribution AUC_t-∞_	significant	insignificant

**Table 6:** Response of the optimized formulation to the stability test.

Month	pH	MS (g ± SD)	DF (g ± SD)	ET (h ± SD)	FT (h ± SD)	Contents (% ± SD) *n* = 3	
						BZN	TIB
0	6.75	32.19 ± 3.28	38.37 ± 2.63	30.04 ± 0.52	>20.0	99.62 ± 0.14	98.10 ± 0.27
0.5	6.73	34.61 ± 4.02	39.13 ± 3.62	>30 h	>20.0	99.54 ± 1.33	99.92 ± 1.66
1	6.78	33.86 ± 2.19	39.58 ± 1.09	>30 h	>20.0	99.87 ± 0.86	99.00 ± 0.98
3	6.78	33. 82 ± 3.67	39.33 ± 1.11	>30 h	>20.0	98.67 ± 0.69	98.28 ± 1.79
6	6.78	33.05 ± 2.33	39.98 ± 2.23	>30 h	>20.0	99.19 ± 0.83	99.48 ± 0.17
Release profile comparison after stability conditions							
Similarity factor (*f*_2_)						70.72	66.19
Dissimilarity factor (*f*_1_)						7.00	10.79

**Table 7:** Statistical analysis of the formulation F10 exposed to stability testing.

Before–after Stability	Mean	Standard Deviation	Standard Error Mean	95% Confidence Interval of the Difference		*t* Value	df	Sig. (2-Tailed)
				Lower	Upper			
BZN	−2.617	2.853	1.078	−5.256	0.022	−2.426	6	0.051
TIB	−3.442	3.355	1.268	−6.545	−0.339	−2.715	6	0.035

## Data Availability

The data presented in this study are available on request from the corresponding author.
